# Lupus Remission: How Do Patient and Physician Perceptions Align?

**DOI:** 10.3390/healthcare14081004

**Published:** 2026-04-11

**Authors:** Chiara Orlandi, Micaela Fredi, Cesare Tomasi, Martina Salvi, Cecilia Nalli, Chiara Bazzani, Liala Moschetti, Ilaria Cavazzana, Franco Franceschini

**Affiliations:** 1 Rheumatology and Clinical Immunology, ASST Spedali Civili of Brescia, ERN-ReCONNET Center, 25123 Brescia, Italy; 2 Department of Clinical and Experimental Sciences, University of Brescia, 25123 Brescia, Italy; 3 Unit of Infectious and Tropical Diseases, ASST Spedali Civili of Brescia, 25123 Brescia, Italy

**Keywords:** patient-reported outcomes, discrepancy, remission

## Abstract

Objective: Clinical remission is a major therapeutic goal in systemic lupus erythematosus (SLE) because of its association with improved long-term outcomes. However, its relationship with patient-reported burden, quality of life, and disease perception remains incompletely understood. This study aimed to evaluate patient-reported outcomes (PROs) in patients with SLE in clinical remission, identify factors associated with impaired health-related quality of life (HRQoL), and assess physician–patient discordance in disease activity perception. Methods: A total of 106 adult patients with SLE in clinical remission according to the definition proposed by Zen et al. were enrolled at a single rheumatology center. Patients were classified into complete remission, clinical remission off corticosteroids, or clinical remission on corticosteroids. Demographic, clinical, and treatment-related data were collected, including organ damage (SLICC-SDI) and disease activity (SLEDAI-2K). Patients completed PRO measures including SF-36, Global Health (GH), pain VAS, STAI-Y1 and STAI-Y2, Zung Depression Scale, Insomnia Severity Index, and HAQ. Disease activity was assessed by both the patient (PGA) and the physician (PhGA); a PGA–PhGA difference >25 mm was considered clinically relevant discordance. Results: Among patients in clinical remission, mild anxiety was observed in 17.1% according to STAI-Y1 and in 27.9% according to STAI-Y2, mild-to-moderate depressive symptoms in 47.1%, and mild insomnia in 25.5%. Of the 106 patients, 24 (22.6%) were in complete remission, 27 (25.5%) in clinical remission off corticosteroids, and 55 (51.9%) in clinical remission on corticosteroids. Patients in clinical remission on corticosteroids showed worse patient-reported outcomes than those in complete remission or clinical remission off corticosteroids. In multivariable analyses, poorer physical HRQoL was independently associated with functional disability, pain intensity, and depressive symptoms, whereas poorer mental HRQoL was independently associated with trait and state anxiety. Clinically relevant physician–patient discordance was observed in 22.6% of the cohort and was almost exclusively driven by higher patient than physician scores. Pain intensity emerged as the most robust independent correlate of discordance. Conclusions: A substantial patient-reported burden may persist in patients with SLE despite clinical remission. Pain, psychological distress, insomnia, and functional disability contribute to impaired HRQoL, while physician–patient discordance appears to reflect a broader mismatch between inflammatory disease control and the patient’s lived experience of illness. These findings support a more comprehensive and patient-centered approach to remission assessment in SLE.

## 1. Introduction

Systemic Lupus Erythematosus (SLE) is a chronic autoimmune disease characterized by a wide range of clinical manifestations and fluctuating disease activity. Optimal management of SLE aims to achieve and maintain clinical remission to prevent organ damage and improve patients’ quality of life. Treat-to-target therapy in SLE (T2T/SLE) identifies clinical remission as one of the main therapeutic goals [1]. It has been demonstrated that clinical remission is associated with better long-term outcomes, especially if achieved early (early remission, within one year of onset) and persists over time [2]. However, clinical remission is not always an easily achievable target, especially when stricter definitions are applied, and it is often difficult to maintain over time. An alternative therapeutic target is the Lupus Low Disease Activity State (LLDAS), which represents a valid option, particularly in the early phases of disease management, as it is more attainable and independently associated with reduced damage accrual [3].

The LLDAS is defined by disease activity and the intake of immunosuppressive drugs. Patients who achieve LLDAS have a significantly reduced frequency of disease flares and organ damage, demonstrating the usefulness of LLDAS as a therapeutic target [4,5,6].

The T2T/SLE strategy has also been introduced to improve patients’ quality of life, in addition to reducing morbidity and mortality. According to the 2019 and 2023 EULAR recommendations, improvement in health-related quality of life (HRQoL), including physical, functional, social, and emotional domains, represents one of the main goals of SLE management, alongside clinical remission or LLDAS [7,8]. Nevertheless, although clinical remission and LLDAS are clearly associated with better clinical outcomes, data regarding the relationship between disease activity and patients’ perceived quality of life remain inconsistent [9,10,11,12,13].

In this context, the present study aimed to investigate the persistence of patient-reported burden in a cohort of patients with SLE in clinical remission, focusing on health-related quality of life, pain, anxiety, depression, insomnia, and disability. We also examined physician–patient discordance in disease activity assessment as one possible expression of this residual burden in order to identify its clinical and psychological correlates.

By integrating clinical data with patient-reported outcomes, this study seeks to broaden the understanding of remission in SLE beyond the absence of overt inflammatory activity, emphasizing that physician-defined remission does not necessarily coincide with patient well-being.

## 2. Methodology

### 2.1. Study Population and Inclusion Criteria

Adult patients with a diagnosis of SLE attending the outpatient clinic of a single rheumatology center between March and July 2021 were enrolled if they were in clinical remission at the time of evaluation according to the definition proposed by Zen et al. [14]. In the present study, remission was assessed at a single time point, namely, at the study visit.

According to the definition proposed by Zen et al. [14], patients were classified into three remission subgroups:

1. Complete remission: no evidence of disease activity either clinically or serologically (SLEDAI-2K = 0), without corticosteroids or immunosuppressive therapy; antimalarials were allowed.
2. Clinical remission off corticosteroids: serologically active but clinically quiescent disease (SACQ; cSLEDAI = 0), without corticosteroids; immunosuppressants and antimalarials were allowed.
3. Clinical remission on corticosteroids: SACQ patients receiving a prednisone-equivalent dose ≤5 mg/day; immunosuppressants and antimalarials were also allowed.

Patients were required to have at least one year of follow-up and be able to read and understand the administered questionnaires. Patients with intellectual disabilities or insufficient knowledge of the Italian language were excluded. Overall, 106 patients fulfilling remission criteria at the study visit were included.

This study was conducted in accordance with the Declaration of Helsinki. Written informed consent was obtained from all participants, and the study protocol was approved by the local ethics committee (approval number: 2333).

### 2.2. Clinical Data Analysis

Clinical data regarding medical history, laboratory findings, and SLE treatments were collected and analyzed. Disease activity (SLEDAI-2K), organ damage (SLICC-SDI), comorbidities, chronic use of NSAIDs or analgesics, antidepressants and/or anxiolytics, corticosteroid treatment, and disease duration were recorded.

To provide additional information on recent historical disease activity, retrospective clinical SLEDAI (cSLEDAI) scores were retrieved when available. A mean historical cSLEDAI was calculated from values recorded at 12, 24, 36, and 48 months before the study visit.

### 2.3. Patient Reported Outcomes (PROs)

Patients assessed their disease activity using a patient global assessment (PGA) on a 0–100 mm visual analog scale (VAS), while physicians provided a corresponding physician global assessment (PhGA). The discrepancy between PGA and PhGA (PGA-PhGA discordance) was calculated, and a difference ≥25 mm was considered discordant, as previously proposed [15].

Patients also rated their pain (VAS pain) and general health (GH) on a 0–100 scale. Several questionnaires were used to gather additional information:

- SF-36 (36-Item Short Form Survey): Assesses health-related quality of life across 8 domains, with scores from 0 to 100. The results can be summarized into physical (PCS) and mental health scores (MCS) [16].
- STAI (State-Trait Anxiety Inventory): Measures anxiety in two forms, state (temporary) and trait (persistent), with scores from 20 to 80.
- ZDS (Zung Depression Scale): Evaluates depression through 20 items, with scores from 20 to 80. Higher scores indicate more severe depression.
- ISI (Insomnia Severity Index): Assesses insomnia severity with scores from 0 to 28, ranging from no insomnia to severe insomnia [17].
- HAQ Disability Index: Measures disability in 8 areas of daily functioning, with scores from 0 to 3, where higher scores indicate greater disability [18].

### 2.4. Statistical Analysis

Continuous variables were expressed as the median and interquartile range (IQR), whereas categorical variables were reported as frequencies and percentages. The distribution of continuous variables was assessed using the Kolmogorov–Smirnov test. Since most variables were not normally distributed, non-parametric analyses were performed.

Comparisons among the three remission subgroups were performed using the Kruskal–Wallis test, followed by Mann–Whitney U tests for post-hoc comparisons. Associations between continuous variables were evaluated using Spearman’s correlation coefficient. Associations between categorical variables were assessed using the chi-square test or Fisher’s exact test, as appropriate.

To identify independent predictors of health-related quality of life, stepwise multiple linear regression analyses were performed using the physical and mental component summary scores of the SF-36 (PCS and MCS) as dependent variables. Candidate predictors included demographic, clinical, treatment-related, and patient-reported variables, namely, age, sex, disease duration, SLICC-SDI, remission subgroup, corticosteroid exposure, anxiolytic and/or antidepressant therapy, pain intensity, depressive symptoms, anxiety scores, insomnia severity, and functional disability.

To investigate factors associated with clinically relevant physician–patient discordance, binary logistic regression analyses were performed using PGA–PhGA discordance >25 mm as the dependent variable. Candidate predictors included demographic factors (age, sex), disease-related variables (disease duration, SLICC-SDI, remission subgroup), treatment-related variables (corticosteroid exposure, corticosteroid dose, anxiolytic and/or antidepressant therapy), and patient-reported outcomes (pain intensity, SF-36 domains, anxiety scores, depressive symptoms, insomnia severity, and functional disability). In addition to the full stepwise model, separate regression models were explored according to variable domains:

(1) demographic variables,
(2) disease-related variables,
(3) treatment-related variables, and
(4) patient-reported outcomes.

Exploratory mediation-oriented analyses were also performed using Hayes’ PROCESS macro for SPSS (version 30.02), following a regression-based approach to test plausible pathways suggested by the main results, including the role of pain and physical function in the relationship between corticosteroid exposure and discordance and the role of depressive symptoms in the association between disease duration and mental health-related quality of life.

A two-sided *p*-value < 0.05 was considered statistically significant. Statistical analyses were performed using IBM SPSS Statistics.

## 3. Results

### 3.1. Cohort Characteristics

Table 1 provides an overview of the 106 patients with SLE included in this study. Most patients were female (87.7%) and Caucasian (98%). The median age at diagnosis was 28 years and the median age at study enrollment was 48 years. The median disease duration was 227 months (approximately 19 years). Thirty-three patients (31.1%) were smokers. The most frequently affected organ systems were mucocutaneous (87.7%) and musculoskeletal (82.1%). Anti-dsDNA antibodies were positive in 94.3% of patients, and anti-ENA and antiphospholipid antibodies were present in 56.6%. Almost all patients had received corticosteroids (97.2%) and hydroxychloroquine (95.3%) during the course of the disease. Additionally, 82.1% had received conventional synthetic DMARDs, 28.3% belimumab, and 62.3% antiplatelet or anticoagulant therapy.

### 3.2. Disease Activity and Remission Subgroups

At enrollment, disease activity was assessed using the SLEDAI-2K index. Because only serological activity was allowed for inclusion, the median SLEDAI-2K score was 0 (IQR: 0–2). According to the remission classification proposed by Zen et al., 24 patients (22.6%) were in complete remission, 27 (25.5%) were in clinical remission off corticosteroids, and 55 (51.9%) were in clinical remission on corticosteroids.

Fifty-seven patients had accrued organ damage, with a median SLICC-SDI score of 1, indicating overall limited damage burden. At the time of the study visit, corticosteroids were being taken by 51.9% of patients, with a median weekly dose of 20 mg. Hydroxychloroquine was used by 78.3% of patients, while azathioprine and mycophenolate mofetil were each used by 12.3%. Other common medications included NSAIDs (22.6%), pain relievers (24.5%), antidepressants (14.2%), and anxiolytic and/or hypnotic therapy (20.8%).

To provide additional information on recent historical disease activity, a mean historical cSLEDAI was calculated for each patient using values recorded at 12, 24, 36, and 48 months before the study visit. This variable was overall low in the cohort (mean ± SD: 0.37 ± 0.79; median 0.0, IQR 0.0–0.25) and did not significantly differ among remission subgroups (Kruskal–Wallis *p* = 0.153). At 12 months before the study visit, 94/106 patients (88.7%) were already in remission, 9 had missing data, and only 3 showed residual disease activity. Considering both the 12- and 24-month timepoints, approximately 80% of the cohort had remained in remission for at least 24 months.

### 3.3. Analysis of PROs

All 106 patients completed the PRO assessment battery, and the results are summarized in Table 2. The median PGA was 10 (IQR: 0–30), whereas the median PhGA was 0 (IQR: 0–0). The median pain score on the visual analog scale was 10 (IQR: 0–30) and the median general health score was 20 (IQR: 10–40), with higher scores indicating worse perceived health.

The SF-36 questionnaire was completed by 104 of 106 patients. General health and vitality were the most impaired domains. The median physical component summary (PCS) was 50 (IQR: 37.5–53) and the median mental component summary (MCS) was 48 (IQR: 38–55).

The median STAI-Y1 and STAI-Y2 scores were 35 and 37, respectively. Mild anxiety was observed in 17.1% of patients according to STAI-Y1 and 27.9% according to STAI-Y2, whereas moderate anxiety was observed in 14.3% and 12.6%, respectively. According to the Zung scale, 47.1% of patients had mild-to-moderate depressive symptoms. Regarding sleep, 61.3% of patients reported no insomnia, while 25.5% had mild insomnia. The median HAQ score was 0 (IQR: 0–0.125; minimum–maximum 0–2.62), indicating overall limited disability.

### 3.4. Comparison Among Remission Subgroups

Since most variables were not normally distributed according to the Kolmogorov–Smirnov test, non-parametric analyses were performed. The Kruskal–Wallis test showed significant differences among remission groups for PGA, PGA–PhGA discordance, depression (Zung scale), insomnia, functional disability (HAQ), General Health, VAS pain, and several SF-36 scales, such as physical activity, bodily pain, and role emotional limitations (all *p* < 0.05). No significant differences were observed for anxiety scores (STAI-Y1 and STAI-Y2), vitality, social functioning, overall mental health, or overall physical health (Table 2).

Post-hoc comparisons using the Mann–Whitney U test showed no significant differences between patients in complete remission and those in clinical remission off corticosteroids. In contrast, when comparing complete remission with clinical remission on corticosteroids, significant differences were observed for PGA (*p* = 0.001), PGA–PhGA discordance (*p* = 0.013), VAS pain (*p* = 0.005), insomnia (*p* = 0.012), bodily pain (*p* = 0.036), and general health perception (*p* = 0.036).

Finally, the comparison between clinical remission off corticosteroids and clinical remission on corticosteroids revealed significant differences for PGA (*p* = 0.002), PGA–PhGA discordance (*p* = 0.005), VAS pain (*p* = 0.016), depression (Zung) (*p* = 0.005), insomnia (*p* = 0.001), HAQ (*p* = 0.034), general health perception (*p* = 0.038), physical activity (*p* = 0.010), bodily pain (*p* = 0.022), and role emotional limitations (*p* = 0.017).

### 3.5. Predictors of HRQoL

Stepwise multiple linear regression analyses were performed to identify predictors of the physical and mental component summary scores of the SF-36. Candidate variables entered into the models included demographic, clinical, treatment-related, and patient-reported measures, namely, age, sex, disease duration, SLICC-SDI, remission subgroup, corticosteroid exposure, anxiolytic and/or antidepressant therapy, pain intensity, depressive symptoms, anxiety scores, insomnia severity, and functional disability.

For the physical component (PCS), the final model included functional disability (HAQ), pain intensity (VAS pain), and depressive symptoms (Zung scale), explaining 57.5% of the variance (adjusted *R*^2^ = 0.562; *F* = 43.336, *p* < 0.001). Higher HAQ scores (*β* = −0.392, *t* = −4.806, *p* < 0.001), greater pain intensity (*β* = −0.385, *t* = −4.704, *p* < 0.001), and higher depression scores (*β* = −0.158, *t* = −2.211, *p* = 0.029) were independently associated with poorer physical health.

For the mental component (MCS), the final model retained trait anxiety (STAI-Y2) and state anxiety (STAI-Y1), explaining 65.1% of the variance (adjusted *R*^2^ = 0.643; *F* = 90.310, *p* < 0.001). Higher trait anxiety (*β* = −0.487, *t* = −3848, *p* < 0.001) and higher state anxiety (*β* = −0.344, *t* = −2.714, *p* = 0.008) were independently associated with worse mental health scores.

### 3.6. Comparison Between Concordant and Discordant Patients

Differences between the disease activity assessments made by patients (PGA) and physicians (PhGA) on a scale from 0 to 100 were calculated. Assessments were considered discordant if the difference was ≥25, as indicated by previous studies [15,19]. Based on this criterion, 82 patients (77.4%) were classified as concordant and 24 (22.6%) as discordant. Among discordant cases, patient scores were almost always higher than physician scores.

In univariate analyses, discordant patients were older and more frequently belonged to the clinical remission on corticosteroids subgroup. They also showed an overall worse patient-reported profile, with higher pain, poorer general health, worse health-related quality of life, greater psychological distress, and higher disability compared with concordant patients (Table 3).

Binary logistic regression analyses were performed using clinically relevant discordance between PGA and PhGA (VAS difference >25 mm) as the dependent variable. In the full stepwise model, higher pain intensity and worse role emotional functioning were independently associated with discordance. Specifically, VAS pain was positively associated with the odds of PGA–PhGA discordance (OR 1.084, 95% CI 1.046–1.123; *p* < 0.001), whereas role emotional limitations were inversely associated (OR 0.983, 95% CI 0.967–0.999; *p* = 0.043). The model showed good fit (Hosmer–Lemeshow *p* = 0.774) and explained 60.8% of the variance (Nagelkerke *R*^2^ = 0.608), with an overall classification accuracy of 89.0%.

Additional domain-specific logistic regression models were performed to explore the contribution of different variable blocks. Among demographic variables, older age was associated with higher odds of discordance (OR 1.053, 95% CI 1.015–1.094; *p* = 0.007). Among disease-related variables, remission subgroup was significant overall (*p* = 0.007), with patients in clinical remission on corticosteroids showing higher odds of discordance compared with those in complete remission (OR 13.143, 95% CI 1.648–104.795; *p* = 0.015). Among treatment-related variables, corticosteroid exposure was independently associated with discordance (OR 6.714, 95% CI 2.106–21.403; *p* = 0.001). However, when patient-reported outcomes were included, pain intensity remained the most robust independent predictor.

A sensitivity analysis using a stricter discordance threshold (≥30 mm) was also performed. Applying this higher cut-off substantially reduced the number of discordant patients, resulting in a marked imbalance between groups and a loss of statistical significance of the associations observed in the primary analysis.

Exploratory mediation-oriented analyses were performed using Hayes’ PROCESS macro for SPSS. Pain intensity was strongly associated with PGA–PhGA discordance, and when pain was included in the model, the association between corticosteroid exposure and discordance was no longer statistically significant, whereas pain remained a strong predictor. In a separate model including the SF-36 role physical domain, both corticosteroid exposure and role physical limitations remained significantly associated with discordance. Finally, depressive symptoms were strongly associated with poorer mental health scores, whereas disease duration was not significant in the corresponding model.

## 4. Discussion

Treat-to-target strategies in SLE aim to achieve and maintain clinical remission in order to prevent organ damage and improve long-term outcomes. However, remission is a multidimensional concept, and its clinical definition does not necessarily reflect the patient’s lived experience of the disease. In this study, we investigated patient-reported burden in a cohort of patients fulfilling a stringent definition of clinical remission, focusing on health-related quality of life, psychological symptoms, disability, and physician–patient discordance. Our findings show that a substantial patient-reported burden may persist despite physician-defined remission, supporting the concept that clinical quiescence does not necessarily coincide with patient well-being. A similar discrepancy has also been observed in recent cohorts, in which patients in a lupus low disease activity state (LLDAS) continued to report significant residual symptoms [20].

Our cohort included 106 patients in remission according to the definition proposed by Zen et al. [14], including 24 patients (22.6%) in complete remission, 27 (25.5%) in clinical remission off corticosteroids, and 55 (51.9%) in clinical remission on corticosteroids.

This distinction proved clinically relevant, as patient-reported burden was not uniformly distributed across remission states. In particular, patients in clinical remission on corticosteroids showed worse scores in several PROs compared with the other subgroups, whereas no major differences emerged between complete remission and clinical remission off corticosteroids. This finding is consistent with previous literature showing that remission achieved without glucocorticoids is associated with significantly better physical quality of life than remission maintained with steroids [21]. In addition, chronic corticosteroid use has emerged as an independent predictor of belonging to a “high symptom burden” cluster, characterized by worse fatigue, mental health, and physical health perception, further confirming that remission is not a homogeneous state from the perspective of perceived well-being [22]. The worst patient-reported outcomes observed in patients in clinical remission on corticosteroids may partly reflect residual treatment burden in addition to residual symptom burden.

The multivariable analyses further clarified which factors were most strongly associated with health-related quality of life in this cohort. For the physical component of the SF-36, functional disability, pain intensity, and depressive symptoms emerged as independent correlates of poorer physical health. This finding suggests that even in the absence of overt clinical activity, physical well-being remains strongly influenced by residual symptom burden and impairment in daily functioning. In particular, pain appears to play a central role, confirming that patient-perceived disease burden in remission is shaped not only by inflammatory activity but also by persistent symptoms that may remain clinically relevant despite disease quiescence. By contrast, the mental component of the SF-36 was primarily associated with anxiety, with both trait and state anxiety independently linked to worse mental health scores. This result is consistent with previous literature showing that psychological distress is a major determinant of mental health-related quality of life in SLE, even when disease activity is controlled [23]. Taken together, these findings support the view that physical and mental aspects of quality of life are driven by partly distinct but interconnected dimensions of burden, with pain, disability, and depressive symptoms contributing more strongly to physical health, and anxiety playing a dominant role in mental well-being.

Longitudinal studies support this strong association between remission and the physical component summary (PCS) but highlight a much weaker or absent relationship with the mental component summary (MCS) [21]. Indeed, while prolonged quiescence may improve physical health, it often appears insufficient to produce substantial improvements in mental health, fatigue, or social functioning [24]. It has also been shown that only very prolonged remission, lasting more than 5 years, has a marked positive impact on several HRQoL domains, including general health and social functioning [25].

These associations should not be interpreted as evidence of causality. For example, the relationship between corticosteroid use and poorer patient-reported outcomes may reflect residual disease burden, greater treatment dependency, or unmeasured aspects of disease severity rather than a direct detrimental effect of corticosteroids per se. Similarly, pain, functional disability, depressive symptoms, and anxiety should be interpreted as correlates or potential determinants of impaired quality of life rather than definitive causal factors. Longitudinal studies will be necessary to clarify the temporal and mechanistic relationships between treatment exposure, symptom burden, and patient-reported outcomes in remission.

Psychological burden remained clinically relevant in our cohort despite remission, and this aspect deserves careful consideration. Although anxiety and depression scores were lower than those reported in unselected SLE populations [26], they remained far from negligible, indicating that symptom control and inflammatory quiescence do not necessarily translate into full psychological well-being. This is consistent with previous studies showing that mood disorders, anxiety, and other non-inflammatory symptoms may persist even in patients with controlled disease activity and substantially contribute to impaired quality of life and worse PROs [23,24,25,27,28].

A particularly relevant aspect of this psychological burden is the discrepancy between the relatively low number of formally diagnosed depressive disorders and the much higher proportion of patients screening positive for depressive symptoms on the Zung questionnaire is particularly noteworthy. While this finding may suggest under-recognition of mood disorders in routine clinical care, it may also reflect the intrinsic limitations of screening instruments in patients with chronic inflammatory disease. The Zung Self-Rating Depression Scale includes several somatic items—such as fatigue, sleep disturbances, and appetite changes—that may overlap with symptoms of SLE itself or with treatment-related effects, particularly corticosteroid exposure. As a result, depressive symptom scores may be inflated, potentially leading to an overestimation of major depressive disorder prevalence.

At the same time, these symptoms may also be underestimated in clinical practice, as clinicians may attribute them to lupus or treatment rather than a coexisting mood disorder. For this reason, patient-reported screening tools should not be interpreted as diagnostic instruments but rather as indicators of possible psychological distress requiring further evaluation. In rheumatology practice, they may serve as useful triage tools to identify patients who could benefit from a more formal psychiatric or psychological assessment. High depression scores in SLE should therefore be interpreted with caution, particularly in the presence of prominent somatic symptoms, without being either automatically equated with major depressive disorder or dismissed as merely disease-related manifestations. Instead, they should prompt a more integrated clinical assessment addressing both psychological and physical contributors to patient-reported burden.

A major limitation of our study is the absence of a specific assessment of fatigue. Fatigue is widely recognized as one of the most prevalent and disabling symptoms in SLE and represents one of the most important patient-reported outcomes, often showing only a weak relationship with objective inflammatory activity. In this sense, the lack of a dedicated fatigue measure may represent the most relevant methodological limitation of the present study. Fatigue and pain have been identified as the main determinants of impairment in daily activities and work productivity, even in patients achieving the LLDAS target [20].

This point is particularly important when interpreting the pattern of patient-reported burden observed in our cohort. The impairment detected in the vitality domain of the SF-36, together with the lower scores in general health perception, may largely reflect the burden of fatigue, which was not directly captured by our assessment tools. Fatigue may also contribute substantially to the persistence of residual burden despite remission, and possibly to the physician–patient discordance observed in a subset of patients. Although the SF-36 vitality domain may partially act as a proxy for fatigue, it cannot fully capture the multidimensional nature of this symptom, which includes physical, cognitive, and emotional components. For this reason, future studies investigating patient-reported outcomes in SLE remission should systematically include validated fatigue-specific instruments, such as the FACIT-Fatigue scale, in order to better characterize its contribution to quality of life, illness perception, and discordance between patient and physician assessments.

Recent historical disease activity was also examined to better contextualize patient-reported burden in remission (48 months before the last evaluation). Although detailed longitudinal data on time to first remission and the full disease course were not systematically available for the entire cohort, nearly 80% of patients had been in remission for at least 24 months. This finding is particularly relevant for interpreting the residual burden observed in remission, as it suggests that the persistence of impaired quality of life, psychological distress, and physician–patient discordance in our cohort cannot be readily explained by higher levels of recent inflammatory disease activity.

Interestingly, this observation differs from previous reports suggesting that prolonged remission may have a favorable impact on health-related quality of life [25,29]. In our cohort, however, recent historical disease activity was low and did not appear to account for differences in patient-reported burden. This observation should be interpreted cautiously, as not all patients had necessarily been in sustained remission throughout the entire 48-month period. Accordingly, our findings do not exclude a beneficial effect of prolonged remission but rather indicate that recent inflammatory control alone may not fully explain the persistence of residual burden.

Taken together, these data support the concept that persistent burden in SLE remission is not merely a residual reflection of recent inflammatory activity but rather the result of a broader interplay of non-inflammatory symptoms, psychological factors, functional impairment, and treatment-related burden.

One of the most relevant findings of our study is that clinically relevant physician–patient discordance was observed in 22.6% of patients, despite all participants fulfilling criteria for clinical remission at the time of evaluation. Although this proportion may appear limited in absolute terms, its persistence in a cohort characterized by low recent inflammatory activity, stable treatment, and, in many cases, remission without corticosteroids or even complete remission is, in our opinion, clinically meaningful rather than negligible.

Our discordance rate was slightly lower than that reported by Yen et al., who observed discrepancies in 28% of an unselected SLE cohort using the same scale and cut-off [30]. However, their population also included patients with active disease, which likely contributed both to the higher overall discordance rate and to the presence of cases in which physicians rated disease activity higher than patients. In contrast, in our remission-only cohort, discordance was almost exclusively driven by higher patient than physician scores. This makes our finding particularly relevant as it suggests that even in the absence of clinically detectable inflammatory activity, a substantial proportion of patients continue to perceive their lupus as active or burdensome. Physician–patient discrepancy has also been reported in other chronic rheumatic diseases. We recently analyzed a cohort of rheumatoid arthritis patients in Boolean remission and found discrepancies in more than 30% of cases, despite the use of a higher PHGA–PGA difference cut-off (30 mm versus 25 mm in the present study) [31].

In this context, discordance should probably not be interpreted simply as disagreement between physician and patient, nor as evidence that the physician is underestimating inflammatory disease activity. Rather, our findings support the interpretation of discordance in remission as a misalignment of focus. Physicians appropriately assess remission on the basis of inflammatory activity and validated clinical indices such as cSLEDAI, whereas patients tend to integrate the broader burden of illness—including pain, fatigue, emotional distress, and functional limitations—into their global assessment of disease. From the patient’s perspective, these symptoms may still represent “active lupus,” even when inflammatory disease is clinically quiescent [32].

This interpretation is supported by our multivariable analyses. Pain emerged as the most robust independent predictor of discordance, while older age, remission subgroup, and corticosteroid exposure were associated with discordance in separate domain-specific models. This finding is well supported by the literature: bodily pain has consistently emerged as one of the most important predictors of clinically relevant discordance since patients tend to incorporate broader aspects of physical and psychological well-being into their global assessment, whereas physicians focus mainly on clinical and laboratory signs of disease activity [30]. Other studies have similarly shown that joint involvement and fibromyalgia are key determinants of this divergence in perspectives [33].

Exploratory regression-based analyses suggested that pain may represent a plausible pathway linking corticosteroid exposure and physician–patient discordance, as the association between corticosteroids and discordance was attenuated after adjustment for pain. In contrast, physical role limitations were also associated with discordance, but the corresponding model did not support a clear mediating effect.

From a clinical perspective, these results highlight an important unmet need. Treat-to-target strategies based exclusively on disease activity indices may fail to capture persistent patient-reported burden even in remission. Systematic assessment of pain, fatigue, psychological well-being, and functional limitations should therefore be considered an essential complement to conventional disease activity evaluation in order to better align therapeutic targets with the patient’s lived experience of illness.

This study has several limitations. First, its cross-sectional design does not allow causal relationships to be inferred; therefore, the associations observed between corticosteroid exposure, pain, psychological symptoms, functional impairment, and physician–patient discordance should be interpreted as correlates or potential determinants rather than causal factors. Second, although the cohort was well characterized, the sample size was relatively limited for subgroup analyses, sensitivity analyses using stricter discordance thresholds, and exploratory mediation-oriented models, which should therefore be considered hypothesis-generating rather than confirmatory. Third, detailed longitudinal data on the full disease course, including time to first remission, were not systematically available for the entire cohort. Although we were able to reconstruct recent historical clinical disease activity over the 48 months preceding study entry, this did not allow a complete assessment of the long-term remission trajectory for all patients. Fourth, fatigue was not assessed with a dedicated validated instrument, representing an important methodological limitation of the study. Fifth, the retrospective collection of part of the clinical data may have led to the underestimation of some comorbidities, including depression and fibromyalgia. Finally, the single-center design and the absence of a control group may limit the generalizability of our findings.

Despite these limitations, our findings provide a detailed characterization of patient-reported burden in a clinically well-defined cohort of patients with SLE in remission. They show that remission, even when defined according to stringent clinical criteria, does not necessarily coincide with preserved quality of life or absence of symptom burden from the patient’s perspective. Overall, these results support a more comprehensive view of remission in SLE, in which inflammatory control remains essential but should be complemented by systematic assessment of patient-reported outcomes in order to better align physician-defined remission with patient-perceived well-being. This patient-centered approach is essential to align therapeutic targets with patient priorities and move toward what some authors have described as “social remission” [20].

## 5. Conclusions

Our study shows that a relevant patient-reported burden may persist in patients with SLE despite clinical remission. Pain, psychological distress, insomnia, and functional disability were associated with poorer health-related quality of life, indicating that remission defined by clinical indices does not necessarily coincide with patient-perceived well-being.

Clinically relevant physician–patient discordance was observed in more than one-fifth of the cohort. In this context, discordance may reflect not simply overestimation by the patient but a misalignment between physician-assessed inflammatory activity and the patient’s broader experience of disease burden.

Overall, these findings support a more comprehensive and patient-centered view of remission in SLE, integrating systematic assessment of pain, psychological well-being, fatigue, and functional status into routine care. Further longitudinal studies are needed to better clarify the determinants of persistent symptom burden in remission.

## Figures and Tables

**Table 1:** Epidemiological, clinical, and serological data; cumulative therapy; and comorbidities.

Characteristic	Total (106)
**Epidemiological data**	
Number of patients (%)	106 (100)
Female (%)	93 (87.7)
Caucasian (%)	104 (98)
Median age (yrs) at diagnosis (IQR)	28 (21–37)
Median age (yrs) at study enrollment (IQR)	48 (41–58)
Median duration (months) of the disease (IQR)	227 (124–330)
Median height (meters) (IQR)	1.7 (1.6–1.7)
Median weight (kg) (IQR)	62 (53.0–71.1)
Median BMI (kg/m^2^) (IQR)	22.8 (20.2–25.8)
Smokers (%)	33 (31.1)
**Organ involvement and serology**	
Musculoskeletal involvement (%)	87 (82.1)
Mucocutaneous involvement (%)	93 (87.7)
Heart involvement (%)	32 (30.2)
Lung involvement (%)	15 (14.2)
Renal involvement (%)	46 (43.4)
Neuropsychiatric involvement (%)	44 (41.5)
Hematological involvement (%)	60 (56.6)
Ocular involvement (%)	53 (50)
Anti-dsDNA (%)	100 (94.3)
ENA (%)	60 (56.6)
Positive Coombs (%)	33 (31.1)
Antiphospholipid antibodies (%)	60 (56.6)
Anti-cardiolipin (aCL)	24 (22.6)
Anti-Beta2 glycoprotein I (β2GPI)	25 (23.6)
Lupus anticoagulant (LA)	25 (23.6)
**Therapy**	
Corticosteroids (%)	103 (97.2)
Hydroxychloroquine (%)	101 (95.3)
cDMARDs (%)	87 (82.1)
Belimumab (%)	30 (28.3)
Antiplatelets/anticoagulants (%)	66 (62.3)
**Comorbidities**	
Depression (%)	11 (10.4)
Fibromyalgia (%)	3 (2.8)

cDMARDs: conventional synthetic disease-modifying antirheumatic drugs.

**Table 2:** Analysis of PROs and comparison among remission subgroups.

PROs	Total (106)	Complete Remission (24)	Clinical Remission off CS (27)	Clinical Remission on CS (55)	*p* Value ^1^	*p* Value ^2^	*p* Value ^3^
PGA median (IQR)	10 (0–30)	5 (0–12.5)	10 (0–15)	20 (10–40)	˂0.001	0.001	0.002
PhGA median (IQR)	0 (0–0)	0 (0–0)	0 (0–5)	0 (0–10)	0.146	0.053	0.794
PGA-PhGA difference, median (IQR)	10 (−10–90)	0 (0–12.5)	0 (0–10)	10 (0–30)	0.004	0.013	0.005
VAS pain, median (IQR)	10 (0–30)	0 (0–20)	10 (0–20)	10 (2.5–50)	0.004	0.005	0.016
GH, median (IQR)	20 (10–40)	10 (10–30)	20 (0–30)	30 (10–50)	0.036	0.036	0.038
SF-36 PCS, median (IQR) †	50 (37.5–53)	51 (42–53)	49 (44.25–55)	48 (30–52)	0.059	0.063	0.051
SF-36 MCS, median (IQR) †	48 (38–55)	52 (41–55.5)	51 (40.25–55)	45 (36–52.75)	0.190	0.113	0.185
PF, median (IQR)	90 (68.8–100)	95 (75–100)	95 (90–100)	85 (55–95)	0.019	0.061	0.010
RP, median (IQR)	100 (25–100)	100 (37.5–100)	100 (50–100)	75 (0–100)	0.181	0.315	0.074
BP, median (IQR)	74 (52–100)	74 (72–100)	82 (7.5–100)	74 (41–84)	0.024	0.036	0.022
GH, median (IQR)	48.5 (32–72.3)	60 (43.5–82)	51.3(33.3–76)	45 (30–67)	0.061	0.025	0.185
VT, median (IQR)	55 (45–80)	70 (52.5–82.5)	60 (45–80)	55 (45–70)	0.189	0.072	0.332
SF, median (IQR)	75 (50–100)	87 (62–100)	87 (62–100)	68.5 (50–87)	0.080	0.085	0.059
RE, median (IQR)	100 (33–100)	100 (33–100)	100 (66–100)	66 (8.25–100)	0.024	0.058	0.017
MH median (IQR)	72 (56–84)	80 (54–88)	72 (64–88)	68 (56–83)	0.355	0.248	0.249
STAI-Y1, range 20–80, median (IQR) ‡	35 (30–47)	33.5 (30.8–40.8)	32 (27–39)	38 (32–47.5)	0.242	0.551	0.089
STAI-Y2, range 20–80, median (IQR)†	37 (30–46)	37 (30–45)	34 (28–43.5)	40 (32–46)	0.345	0.439	0.161
Zung test, range 20–80, median (IQR) †	34.5 (29–43)	32.5 (28.3–42.8)	31 (25–39)	38 (32–44.5)	0.011	0.063	0.005
Insomnia Severity Index, median (IQR)	6 (2–12)	4 (1.5–8.75)	3 (0.5–7.5)	7 (4.25–14)	0.001	0.012	0.001
HAQ, median (IQR)	0 (0–0.125)	0 (0–0.03)	0 (0–0)	0 (0–0.5)	0.046	0.083	0.034

CS = corticosteroids; PhGA = Physician Global Assessment of Disease Activity; PGA = Patient Global Assessment of Disease Activity; GH = global health; VAS = visual analog scale; PF = physical functioning; RP = role physical; BP = bodily pain; GH = general health; VT = vitality; SF = social functioning; RE = role emotional; MH = mental health; SF-36 PCS = Short Form 36 Physical Component Summary; SF-36 MCS = Short Form 36 Mental Component Summary; STAI = State-Trait Anxiety Inventory; HAQ = Health Assessment Questionnaire. † The questionnaire was completed by 104 patients out of 106. ‡ The questionnaire was completed by 105 patients out of 106. ^1^ Differences among the three groups were analyzed with the Kruskal–Wallis test. ^2^ Differences among patients in complete remission and clinical remission on CS were evaluated with the Mann–Whitney U test. ^3^ Differences among patients in clinical remission off CS and clinical remission off CS were evaluated with the Mann–Whitney U test. No significant differences were found comparing patients in complete remission and clinical remission off corticosteroids.

**Table 3:** Demographic, clinical, and therapeutic characteristics and PRO results of the two groups.

	Total (106)	Concordant (82)	Discordant (24)	*p* Value
**Demographic characteristics**				
Age at evaluation (median, IQR)	48 (42–58)	46 (39–57)	58 (49–62)	0.004
Female gender	93 (8.7)	72 (82.8)	21 (87.5)	1
Disease duration (months, median, IQR)	227 (125–328)	215 (118–328)	245 (194–321)	0.26
Smokers	33 (31.1)	25 (30.5)	8 (33.3)	0.81
**Clinical characteristics**				
Skin involvement	87 (82.1)	69 (84,2)	18 (75)	0.37
Musculoskeletal involvement	93 (87.7)	71 (86.6)	22 (91.7)	0.73
Cardiac involvement	32 (30.2)	23 (28.1)	9 (37.5)	0.45
Lung involvement	15 (14.2)	11 (13.4)	4 (16.7)	0.74
Renal involvement	46 (43.4)	40 (48.8)	6 (25)	0.060
Neurological involvement	44 (41.5)	32 (39.0)	12 (50)	0.37
Hematological involvement	60 (56.6)	49 (59.8)	11 (45.8)	0.25
Gastrointestinal involvement	3 (2.8)	2 (2.4)	1 (4.2)	0.54
Ocular involvement	53 (50)	40 (48.8)	13 (54.2)	0.82
SLICC-SDI (median, IQR)	1 (0–1)	1 (0–1)	1 (0–1)	0.86
**Therapy**				
CR off corticosteroids	51 (48.1)	47 (57.3)	4 (16.7)	<0.001
Complete remission	20 (18.9)	19 (23.2)	1 (4.2)	0.039
Weekly CS dose, mg *	20 (15–25)	17.5 (11.3–25)	25 (16.9–29.4)	0.051
Anxiolytic/hypnotic therapy	22 (20.8)	14 (17.1)	9 (37.5)	0.048
Antidepressant therapy	15 (14.2)	9 (11.0)	6 (25)	0.10
**PROs**				
PGA [0–100], median (IQR)	10 (0–30)	10 (0–10)	50 (37.5–50)	<0.001
PhGA [0–100], median (IQR)	0 (0–0)	0 (0–0)	0 (0–2.5)	0.81
GH [0–100], median (IQR)	20 (10–40)	10 (10–30)	50 (35–60)	<0.001
VAS-pain [0–100], median (IQR)	10 (0–30)	10 (0–20)	50 (40–60)	<0.001
SF-36 PCS, median (IQR) †	50 (37.5–53)	51 (44–54)	30 (27.5–39)	<0.001
SF-36 MCS, median (IQR) †	48 (38–55)	51 (40–55)	40 (36–48.5)	0.015
PF, median (IQR)	90 (68.8–100)	95 (75–100)	70 (35–82.5)	<0.001
RP, median (IQR)	100 (25–100)	100 (50–100)	0 (0–50)	<0.001
BP, median (IQR)	74 (52–100)	74 (72–100)	41 (30–46)	<0.001
GH, median (IQR)	48.5 (32–75.3)	60 (37–76)	35 (27.5–46)	<0.001
VT, median (IQR)	55 (45–80)	65 (50–80)	50 (42.5–55)	0.007
SF, median (IQR)	75 (50–100)	87 (62–100)	62 (50–68.5)	<0.001
RE, median (IQR)	100 (33–100)	100 (66–100)	33 (0–66)	<0.001
MH, median (IQR)	72 (56–84)	76 (60–88)	68 (54–74)	0.073
STAI-Y1, range 20–80, median (IQR) ‡	35 (30–47)	33 (28.3–45.5)	42 (36.5–49.5)	0.013
STAI-Y2, range 20–80, median (IQR) †	37 (30–46)	35 (29–43.3)	42 (36–46)	0.021
Zung Test, range 20–80, median (IQR) †	34.5 (29–43)	33 (27–43)	39 (35.5–44.5)	0.008
Insomnia severity index, median (IQR)	6 (2–12)	4 (1–9)	9 (6.8–14.3)	<0.001
HAQ, median (IQR)	0 (0–0.1)	0 (0–0)	0.38 (0–0.6)	<0.001

CR = clinical remission; CS = corticosteroids; PhGA = Physician Global Assessment of Disease Activity; PGA = Patient Global Assessment of Disease Activity; GH = global health; VAS = visual analog scale; PF = physical functioning; RP = role physical; BP = bodily pain; GH = general health; VT = vitality; SF = social functioning; RE = role emotional; MH = mental health; SF-36 PCS = Short Form 36 Physical Component Summary; SF-36 MCS = Short Form 36 Mental Component Summary; STAI = State-Trait Anxiety Inventory; HAQ = Health Assessment Questionnaire. * The data related to the weekly dose of corticosteroids were calculated exclusively in patients who used them: 35 patients among the “concordant” and 20 among the “discordant”, for a total of 55 patients out of 106. † The questionnaire was completed by 104 patients out of 106. ‡ The questionnaire was completed by 105 patients out of 106.

## Data Availability

The data supporting the findings of this study are not publicly available due to patient privacy and ethical restrictions.
